# Evaluating therapeutic effects of exoskeletons and FES in SCI: integrative review of the literature

**DOI:** 10.1038/s41393-025-01085-x

**Published:** 2025-05-29

**Authors:** Rachel Y. Kim, Olivia M. Biller, M. J. Mulcahey

**Affiliations:** 1 Center for Outcomes and Measurement, Jefferson College of Rehabilitation Sciences, Thomas Jefferson University, Philadelphia, USA; 2 Johnson & Johnson, Raritan, USA

**Keywords:** Outcomes research, Therapeutics

## Abstract

**Background:**

Functional electrical stimulation and exoskeletons provide direct functional ability but may also have therapeutic effects that improve function when they are turned off or removed.

**Objective:**

This integrative review aimed to identify functional clinical outcome assessments used to assess therapeutic effects in rehabilitation technologies for persons with spinal cord injuries, to examine the National Institute of Neurological Disorders and Stroke Common Data Elements recommendation level for SCI for each COA, and determine which COAs distinguish between recovery of function and function from compensation.

**Methods:**

A literature search identified interventional SCI studies using FES and exoskeletons (*n* = 1006). Text screens resulted in a sample (*n* = 56) organized by level of evidence, COAs with their measurement properties, type of intervention with involved limbs, the NINDS CDE recommendation level, and if the COAs distinguished recovery from compensation.

**Results:**

56 articles met inclusion criteria. 31 studies involved exoskeletons, 23 studies involved FES, and 2 studies involved both FES and exoskeleton. Within those 56 articles, 38 COAs were identified across all studies, including different versions of the same COA as separate measures. Of these 38 COAs, 24 were PerfOs and 7 were PROs. The most used COAs did not differentiate recovery from compensation. However, 3 COAs were identified as able to discriminate recovery from compensation.

**Conclusions:**

Studies on FES and exoskeletons in SCI have precedent to examine therapeutic effects using a variety of functional COAs. Clinical trials in SCI would benefit from COAs with interval scales that assess therapeutic effects that differentiate between recovery and compensation.

## Introduction

### Background

Functional electrical stimulation (FES) and exoskeletons are rehabilitation technologies used to restore functional abilities following spinal cord injury (SCI). FES is the stimulation of two or more muscles to produce a functional FES-movement pattern. When FES is off, the movement pattern is no longer there. Whereas an exoskeleton is an external device (passive or with motor) that provides a functional movement pattern. Without the exoskeleton movement is no longer there. Clinical trials may quantify the immediate benefits that people may experience while wearing exoskeletons and FES systems, as well as the therapeutic effects, that are sustained when the devices are not utilized.

Therapeutic effects of FES and exoskeletons when the devices are turned off or removed may include recovery of movement, increase in muscle strength, improvement in physical functioning and interjoint coordination, among other outcomes [1–3]. Although the assessment of recovery of movement and muscle strength is relatively straightforward, assessment of improvements in physical functioning in SCI is a bit more challenging due to shortcomings in existing clinical outcome assessments (COA), including the ordinal nature of most response scales, presence of both ceiling and floor effects, multi-dimensional constructs, and inability to distinguish between motor recovery and compensatory movement strategies used for function [4–6]. The ability of a COA to distinguish between recovery and compensation is important when assessing therapeutic effects of devices. Owing to the number of clinical trials of FES and exoskeletons that are interested in not only the immediate benefits of FES and exoskeletons while they are used but also the potential therapeutic effects they have, it is important to understand what COAs are currently used to assess therapeutic effects and the degree to which they distinguish between recovery of movement and compensatory movement strategies used for function.

Moreover, most measures of physical function utilized in clinical trials are ordinal and multidimensional. The use of ordinal scales is suboptimal for clinical trials because the differences between item scores are unknown or unequal, whereas interval scales offer equal, known, and objectively scored differences between items [7]. There is a need for a performance-based SCI clinical trial outcome measure that has the measurement properties recommended for clinical trials [7–9].

### Study aim

The aim of this review was to examine COAs that are used to measure the therapeutic effects of exoskeletons as an initial step to determining the need for a new COA that evaluates movement and distinguishes from compensation. To our knowledge, there were no other reviews addressing our guiding questions, or similar questions.

The guiding questions were as follows:

1. What COAs are used in intervention studies that test the therapeutic effects of FES only, exoskeletons only, and combined FES/exoskeletons in SCI for upper and lower limbs?
2. What are the National Institute of Neurological Disorders and Stroke SCI Common Data Elements (NINDS CDE) recommendation levels for each COAs?
3. Among the COAs identified, which ones distinguish between recovery of movement and compensation strategies used for function?

## Methods

### Study design

An integrative review methodology was used to identify, analyze, and synthesize the results from studies. An integrative review provides a synthesis of content knowledge through a systematic process of preparing a guiding question(s), searching the literature, obtaining and analyzing content of manuscripts, discussing results, and presenting the review for application [10]. The inclusion of studies using designs beyond the randomized controlled trial provides a comprehensive understanding of the literature and captures emergent phenomena and trends in the field [10, 11].

### Search strategy

**Fig. 1:**
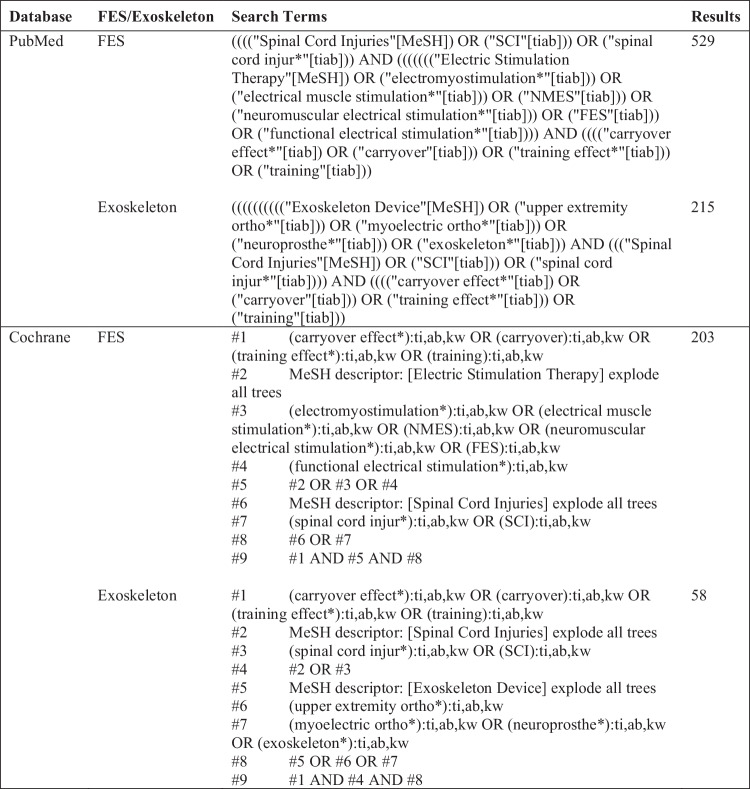
Search Strategy: Medical Subject Heading terms.

This literature review was conducted by two occupational therapy (OT) doctoral students with consultation from a library scientist, using the PubMed and Cochrane Library databases. The keywords that were used within the search strategy were “spinal cord injuries”, “functional electrical stimulation” or “exoskeleton”, and “training effect”. The search was completed using the databases’ thesaurus and relevant Medical Subject Heading terms (Fig. 1).

Two OT doctoral students completed the search and initial selection of studies using the following inclusion criteria: peer-reviewed research; English-language full-text publication; human studies; at least 50% of study participants had SCI; intervention was FES and/or exoskeleton; intervention involved repeated training with FES/Exoskeleton; use of COAs to assess therapeutic effect of FES/exoskeleton on either or both the upper and lower extremity; and studies with a primary or secondary study endpoint being motor and/or physical function. The students collaborated with a research librarian throughout the integrative review process. Studies were excluded if the interventions were not clearly defined, used electrical stimulation only as an exercise modality, and if COAs were only administered to evaluate the immediate effects of FES, exoskeleton or FES/exoskeleton and not to evaluate the therapeutic effects when the devices were off. Grey literature (dissertation, thesis), abstracts, review papers, position papers, and white papers were excluded as well. There were no limits on the date range.

**Fig. 2:**
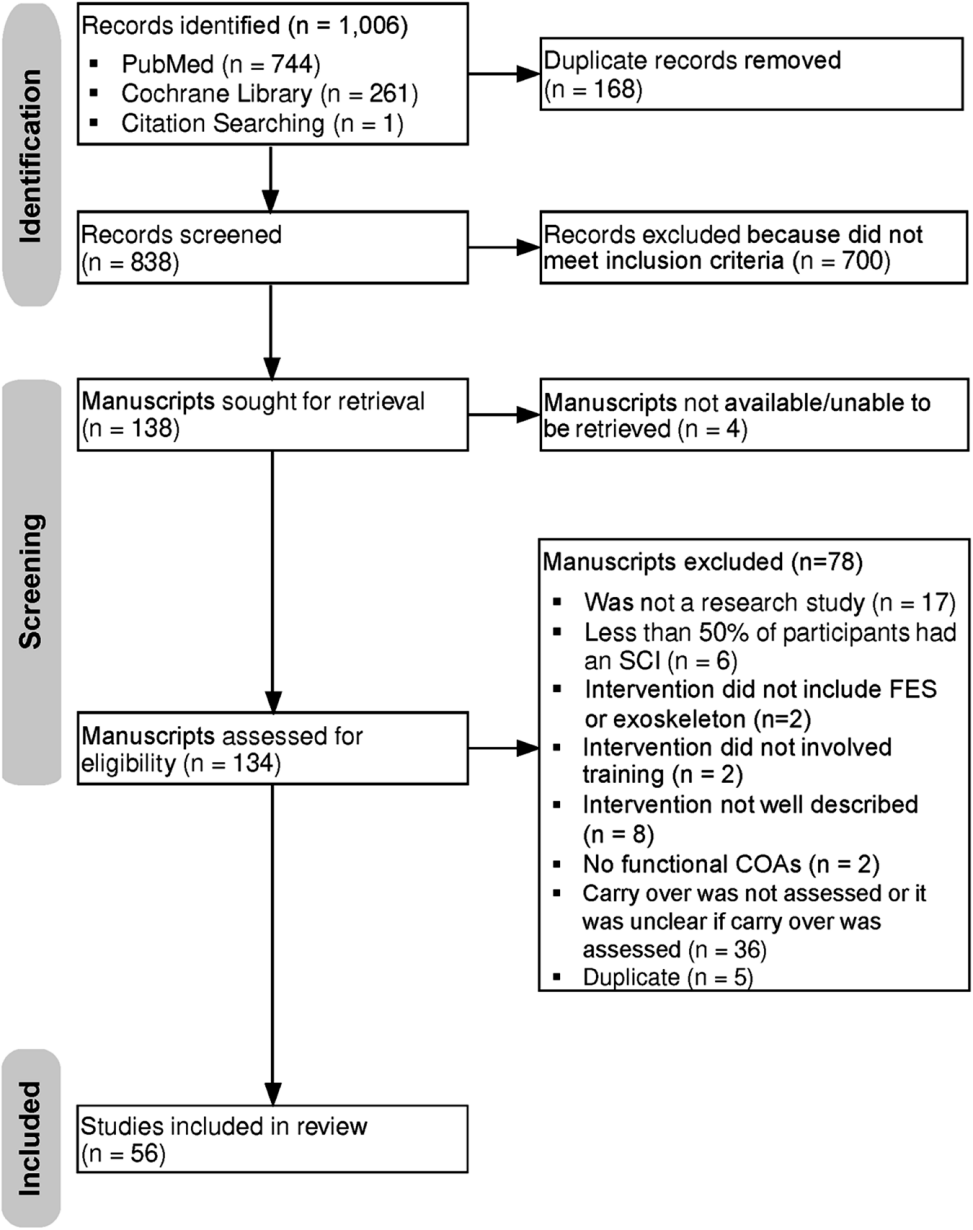
PRSIMA Flowchart.

As illustrated in the PRISMA flowchart (Fig. 2), 1006 manuscripts were identified in PubMed (*n* = 744), Cochrane Library (*n* = 261), and by Citation Searching (*n* = 1) with 168 duplicate records removed. The two OT doctoral students, blinded to each other’s assessment, separately performed a title and abstract screen on 838 records, then met to determine consensus on articles that did not meet agreement, resulting in 700 records failing the initial screen with 138 records advancing to full text screen (Fig. 2). Of the 138 articles, 134 articles could be retrieved for the subsequent full-text screen.

The OT doctoral students were not blinded during the full-text screen but independently reviewed the articles, met to obtain consensus, and reviewed the data from one another’s work. During the final screen for eligibility, manuscripts were excluded if: they were a duplicate with another included manuscript; did not describe COAs that assessed motor or physical function; had poorly described interventions or if the intervention did not involve FES or exoskeleton and did not meet one or more of the inclusion criteria. A total of 56 manuscripts met the inclusion for full review and data extraction.

### Analysis

The level of evidence of studies was defined according to the Oxford Centre for Evidence-Based Medicine Levels of Evidence Working Group [12]. To examine the three guiding questions for this review, COAs were organized by type of COA – namely performance-based outcome (PerfO) and patient-reported outcome (PRO) measures. PerfOs are administered by a trained rater to measure participant functions/functional ability through participant completion of standardized tasks that must be performed according to specific instructions and scored with standardized guidelines (e.g., gait speed, word recall memory test), whereas PROs are completed by the participant or by an interviewer filling in the participant’s responses without any changes or interpretation to better understand a participant’s perspective or experience (e.g., rating scales, counts of events) [13]. The COAs were further examined for level of measurement (nominal, ordinal, interval, ratio), ability to distinguish between motor recovery and compensatory motor strategies, and the NINDS SCI CDE designation. The Rehab Measures Database (RMD) and Physiopedia were used as primary resources to garner information about the measurement construct of COAs [14, 15]. If these databases did not have sufficient information, peer-reviewed manuscripts and Stroke Engine were used as supplements.

If it was unclear whether the COA discriminates between motor recovery and compensation, the following decisions adopted were: (1) patient-reported outcomes (PROs) do not differentiate; (2) performance-based outcomes (PerfOs) that only evaluated “time” (e.g., to walk, to complete task) do not differentiate; (3) for PerfOs that evaluated constructs other than “time”, the administration guidelines, (when available) test items, and response categories were examined, and discussed until consensus was reached among the authors; and (4) if there was insufficient information to confirm the ability to differentiate between recovery and compensation, the COA was marked as “not enough information”.

## Results

Of the 56 studies that met inclusion criteria, 16 studies were level II (randomized trial or observational study with dramatic effect), 24 studies were level III (non-randomized controlled cohort/follow-up study), and 16 studies were level IV (case-series case-control or historically controlled studies) [12]. There were 13 studies on upper limb, 42 studies on lower limb, and 1 study that focused on both the upper and lower limb (see supplementary material). Further, 31 studies involved exoskeletons, 23 studies involved FES, and 2 studies involved both FES and exoskeleton (Table 1). There were 38 COAs identified across all studies, including multiple versions of the same COA that were recorded as separate measures (for example, Walking Index for Spinal Cord Injury I [WISCI I] and Walking Index for Spinal Cord Injury II [WISCI II] were considered two separate COAs) (Table 1). Of these 38 COAs, 24 were PerfOs and 7 were PROs. One COA (Spinal Cord Independence Measure [SCIM]) that has been validated as a PRO and PerfO was administered as a PerfO in 2 studies and PRO in 12 studies.

**Table 1:** Total COAs identified across all studies (*n* = 38).

Functional Outcome Measure	Measurement Construct	Measurement Level	Mode of Admin reported in study	# FES studies	# Exo studies	Both
10 Meter Walk Test (10MWT)	Gait speed	Ratio	PerfO	7	19	2
2 min Walk Test (2MWT)	Endurance	Ratio	PerfO	2	1	1
5 Meter Walk Test (5MWT)	Gait speed	Ratio	PerfO	1	0	0
6 min Walk Test (6MWT)	Endurance	Ratio	PerfO	6	13	1
Action Research Arm Test (ARAT)	Upper limb function	Ordinal	PerfO	1	3	0
Modified Action Research Arm Test (MARAT)	Upper limb function	Ordinal	PerfO	1	0	0
Activity-Based Balance Level Evaluation (ABLE)	Balance	Ordinal or Interval	PerfO	1	0	0
AuSpinal Test of Hand Function (AuSpinal)	Unilateral hand function	Nominal (for item subcomponents scored 0–2 based on ability or inability to complete subcomponent); Ordinal (for item subcomponents scored 0–4)	PerfO	1	0	0
Barthel Index (not reported) (BI)	Functional independence during activities of daily living	Nominal (for items scored 0–1); Ordinal (for items scored 0–2 or 0–3)	Not Reported	1	0	0
Modified Barthel Index (not reported) (MBI)	Functional independence during activities of daily living	Nominal (for items scored 0–1); Ordinal (for items scored 0–2 or 0–3)	Not Reported	0	1	0
Berg Balance Scale (BBS)	Balance	Ordinal	PerfO	2	6	0
Box and Blocks Test (BBT)	Unilateral manual dexterity	Ratio	PerfO	2	1	0
Canadian Occupational Performance Measure (COPM)	Performance and satisfaction with activities	Ordinal	PRO	1	0	0
Capabilities of the Upper Extremity Test (CUE-T)	Limitations in physical function	Ordinal	PerfO	1	0	0
Chedoke Arm and Hand Activity Inventory (CAHAI)	Upper limb function	Ordinal	PerfO	1	0	0
Functional Ambulation Category (FAC)	Functional ambulation	Ordinal	PerfO	0	1	0
Functional Independence Measure (not reported) (FIM)	Level of independence in ADLs	Ordinal	Not Reported	2	6	0
Modified Functional Reach Test (MFRT)	Dynamic balance	Ratio	PerfO	0	2	0
Goal Attainment Scale (GAS)	Extent to which a goal is achieved	Ordinal	PRO	1	0	0
Graded Redefined Assessment of Strength Sensibility and Prehension (GRASSP)	Sensorimotor and prehension	Ordinal	PerfO	2	3	0
Grasp and Release Test (GRT)	Unilateral hand function	Ratio	PerfO	1	0	0
Jebsen-Taylor Hand Function Test (JTHFT)	Unimanual hand function	Ratio	PerfO	1	4	0
Lawton Instrumental Activities of Daily Living Scale	Functional independence with instrumental activities of daily living	Ordinal	PRO	1	0	0
Mini Balance Evaluation Systems Test (Mini BESTest)	Dynamic balance	Ordinal	PerfO	1	0	0
Motor Activity Log (MAL)	Upper limb function	Ordinal	PRO	0	1	0
Nine Hole Peg Test (9HPT)	Hand dexterity	Ratio	PerfO	1	0	0
Patient’s Global Impression of Change Scale (PGIC)	Strength; Gait function (2) - Efficacy of treatment	Ordinal	PRO	0	1	0
Rancho Los Amigos Gait Analysis Assessment	Gait quality	Nominal	PerfO	1	0	0
Reintegration to Normal Living Index (RNLI)	Reintegration into activities	Ordinal	PRO	1	0	1
Spinal Cord Injury Functional Ambulation Inventory (SCI-FAI)	Functional walking ability	Nominal (weight shift, step width, foot contact); Ordinal (step rhythm, step height, step length, assistive devices, walking mobility); Ratio (2-min walk test)	Multi-modal: PerfO & PRO	0	0	1
Spinal Cord Independence Measure (not reported) (SCIM)	Functional status with activities of daily living Looks at self-care (6 items), respiration & sphincter management (4 items), and mobility (9 items)	Ordinal	Not Reported	6	0	0
Spinal Cord Independence Measure-II (not reported) (SCIM-II)	Functional status with activities of daily living Looks at self-care (6 items), respiration & sphincter management (4 items), and mobility (9 items)	Ordinal	Not Reported	0	1	1
Spinal Cord Independence Measure-III (PRO) (SCIM-III)	Functional status with activities of daily living Looks at self-care (6 items), respiration & sphincter management (4 items), and mobility (9 items)	Ordinal	PRO	1	0	1
Spinal Cord Independence Measure-III (not reported) (SCIM-III)	Functional status with activities of daily living Looks at self-care (6 items), respiration & sphincter management (4 items), and mobility (9 items)	Ordinal	Not Reported	0	4	0
Timed Up and Go (TUG)	Gait function	Ratio	PerfO	1	10	2
Toronto Rehabilitation Institute Hand Function Test (TRI-HRT)	UE manipulation and grip force	Ordinal (manipulation, strength for blocks); Ratio (for cylinder, credit card, and wooden bar)	PerfO	1	0	0
Walking Index for Spinal Cord Injury (WISCI)	Level of assistance during walking	Ordinal	PerfO	0	1	0
Walking Index for Spinal Cord Injury-II (WISCI-II)	Level of assistance during walking	Ordinal	PerfO	2	12	0

*ABLE* activity-based balance level evaluation, *ARAT* action research arm test, *AuSpinal* AuSpinal test of hand function, *BBS* berg balance scale, *BBT* box and blocks test, *BI* barthel index, *CAHAI* chedoke arm and hand activity inventory, *COPM* Canadian occupational performance measure, *CUE-T* capabilities of the upper extremity test, *FAC* functional ambulation category, *FIM* functional independence measure, *GAS* goal attainment scale, *GRASSP* graded redefined assessment of strength sensibility and prehension, *GRT* grasp and release test, *JTHFT* jebsen-taylor hand function test, *MARAT* modified action research arm test, *MBI* modified barthel index, *MFRT* modified functional reach test, *Mini BESTest* mini balance evaluation systems test, *MAL* motor activity log, *PGIC* Patient’s global impression of change scale, *RNLI* reintegration to normal living index, *SCI-FAI* spinal cord injury functional ambulation inventory, *SCIM* spinal cord independence measure, *SCIM-II* spinal cord independence measure-II, *SCIM-III* spinal cord independence measure-III, *TUG* timed up and go, *TRI-HRT* Toronto rehabilitation institute hand function test, *WISCI* walking index for spinal cord injury, *WISCI-II* walking index for spinal cord injury-II, *2MWT* 2 min walk test, *5MWT* 5 meter walk test, *6MWT* 6 min walk test, *9HPT* 9 hole peg test, *10MWT* 10 meter walk test.

**Fig. 3:**
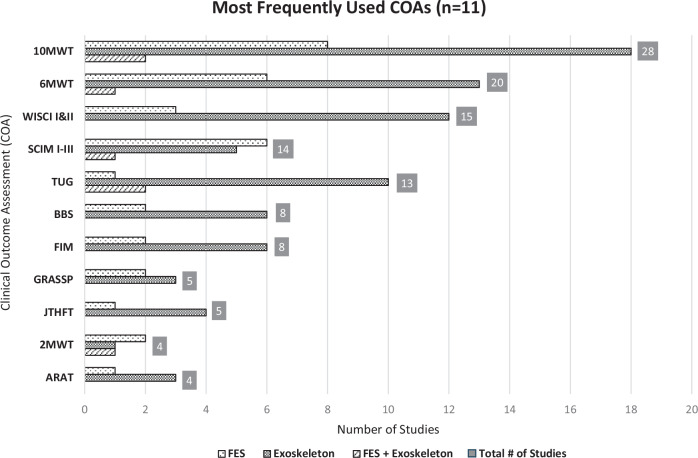
Most Frequently Used COAs (*n* = 11). Other COAs were used in ≤ 3 studies (out of 56 studies). ARAT action research arm test, 2MWT 2 minute walk test, JTHFT jebsen-taylor hand function test, GRASSP graded redefined assessment of strength sensibility and prehension, FIM functional independence measure, BBS berg balance scale, SCIM I-III spinal cord independence measure I-III, TUG timed up and go, WISCI I&II walking index for spinal cord injury I&II, 6MWT 6 minute walk test, 10MWT 10 meter walk test.

Of the 38 COAs, 11 (28.95%) were used in more than one study (Fig. 3). Seventeen (44.74%) of the 38 COAs were included in the NINDS SCI CDE, with only 4 designated as supplemental, highly recommended, and the remaining as supplemental or exploratory (Table 2). Only 3 of the 38 (7.89%) COAs either disallows compensation (Capabilities of the Upper Extremity Test [CUE-T]), or accounts for compensation in scoring (Graded Redefined Assessment of Strength Sensibility and Prehension [GRASSP], Spinal Cord Functional Ambulation Inventory [SCI-FAI]), all ranking supplemental on the NINDS CDE (Table 2).

**Table 2:** COAs recommended by the NINDS CDE.

COA	Measurement Construct	Scale	PerfO or PRO	Differentiates Compensation	NNIDS CDE for SCI Designation
10 Meter Walk (10MWT)	Gait speed	Ratio	PerfO	No	Supplemental – Highly Recommended
2 min Walk (2MWT)	Endurance	Ratio	PerfO	No	Supplemental – Highly Recommended
6 min Walk (6MWT)	Endurance	Ratio	PerfO	No	Supplemental – Highly Recommended
Spinal Cord Independence Measure (SCIM-III)	Functional Status with activities of daily living and mobility	Ordinal	PerfO and PRO	No	Supplemental – Highly Recommended
Berg Balance Scale (BBS)	Balance	Ordinal	PerfO	No	Supplemental
Capabilities of the Upper Extremity Test *(CUE-T)	Limitations of physical function	Ordinal	PerfO	Yes	Supplemental
Graded Redefined Assessment of Strength Sensibility and Prehension (GRASSP)	Sensorimotor and prehension	Ordinal	PerfO	Yes	Supplemental
Jebsen-Taylor Hand Function Test (JTHFT)	Unilateral hand function	Ratio	PerfO	No	Supplemental
Timed Up and Go (TUG)	Gait function	Ratio	PerfO	No	Supplemental
Walking Index for Spinal Cord Injury I&II (WISCI-I-II)	Assistance during walking	Ordinal	PerfO	No	Supplemental
Canadian Occupational Performance Measure (COPM)	Performance and satisfaction with activities	Ordinal	PRO	No	Supplemental
Spinal Cord Injury Functional Ambulation Inventory (SCI-FAI)	Functional walking ability	Nominal, Ratio, or Ordinal	PRO with 1 PerfO item	Yes	Supplemental
Grasp and Release Test (GRT)	Unilateral hand function	Ratio	PerfO	No	Exploratory
Mini Balance Evaluation Systems Test (Mini BESTest)	Dynamic balance	Ordinal	PerfO	No	Exploratory
Nine Hole Peg Test (9-HPT)	Hand dexterity	Ratio	PerfO	No	Exploratory
Toronto Rehabilitation Institute Hand Function Test (TRI-HFT)	Hand function and grip force	Ordinal, Ratio	PerfO	No	Exploratory

**NINDS CDE for SCI Designation:** Exploratory: data element requires further validation but may fill current gaps in the CDEs and/or substitute for existing CDE once validation is complete; Supplemental: data element which is commonly collected in clinical research studies but whose relevance depends upon study design or type of research involved; Supplemental – Highly Recommended: data element is essential based on certain conditions or study types in clinical research studies. They have been used and validated in the disease area and are strongly recommended for the disease condition, study type, or design.

*BBS* berg balance scale, *COPM* Canadian occupational performance measure, *CUE-T* capabilities of the upper extremity test, *GRASSP* graded redefined assessment of strength sensibility and prehension, *GRT* grasp and release test, *JTHFT* jebsen-taylor hand function test, *Mini BESTest* mini balance evaluation systems test, (*SCI-FAI*) spinal cord injury functional ambulation inventory, *SCIM-III* spinal cord independence measure, *TRI-HFT* Toronto rehabilitation institute hand function test, *TUG* timed up and go, *WISCI-I-II* walking index for spinal cord injury I&II, *2MWT* 2 min walk test, *6MWT* 6 min walk test, *9HPT* 9 hole peg test, *10MWT* 10 meter walk test.

When considering the most frequently used COAs, the studies evaluating FES and exoskeleton systems often utilized a combination of walking tests and COAs. Studies that focused on assessing therapeutic effects of exoskeleton systems for walking tend to use a combination of walking tests and COAs (Timed Up and Go [TUG], WISCI I & II, 6-min walk test [6MWT], 10 meter walk test [10MWT]). Those studies that only studied FES systems included SCIM I-III more frequently than studies that examined exoskeleton systems or studies that examined both FES and exoskeleton.

## Discussion

This integrative review described COAs used in SCI studies to evaluate therapeutic effects of FES and exoskeleton interventions. Of the 38 COAs identified across studies, the majority (23/38 or 60.5%) were PerfO. PerfOs may be more appropriate for measuring physical function in the context of SCI than PROs as they include a direct assessment of the participant performing certain standardized tasks and may distinguish recovery of function from compensation, albeit they may not measure a meaningful aspect of health or have as much relevance in real-world situations. Although PROs are often used to measure effectiveness of an intervention, they provide a subjective assessment from the participants on their experiences and functioning without interpretation from anyone else [16, 17]. PROs may not capture therapeutic effects of FES or exoskeleton on movement and movement recovery, albeit their relevance to real world experiences.

It is important to note that for studies with walking as an endpoint of interest, many use the same COA. Specifically, most studies utilized 10MWT (28/56 or 50% of studies), 6MWT (20/56 or 35.7% of studies), TUG (13/56 or 23.2% of studies) or the WISCI-II (15/56 or 26.8% of studies). Except for the WISCI, the walking tests are on a ratio level scale, and designated by NINDS CDE as supplemental, highly recommended (10MWT, 6MWT) or supplemental (TUG). The WISCI-II is also designated as supplemental, however uses an ordinal scale, where the difference between WISCI levels is unknown. COAs on an interval and ratio level of measurement are more precise because they are quantifiable with known differences between possible scores [7]. Given the endorsement of NINDS for the 10MWT, 6MWT, and TUG, it is highly recommended that they are considered when selecting COAs to assess therapeutic effects of FES and exoskeletons on walking.

Unlike walking, there are no COAs that are consistently and repeatedly used to measure endpoints of upper extremity function or physical function. SCIM (versions I-III) was used in 14 of the 56 studies (25%) and is designated as supplemental by NINDS SCI CDE but has known limitations that diminish its effectiveness to detect therapeutic effects [18–20]. The Jebsen-Taylor Hand Function Test (JTHFT), GRASSP, and Box and Blocks Test (BBT) were used in five (8.9%), five (8.9%), eight (14.3%) of the 56 studies, respectively, and two (JTHFT and GRASSP) are designated by NINDS CDE as supplemental. All three are narrow in scope (hand function/grasp/release) and have not been widely adopted. Recommendations for selection of COAs for measuring therapeutic effect on upper extremity and physical function are needed.

Only 44.74% of the COAs had NINDS SCI CDE designation. Four (10MWT, 2MWT, 6MWT, SCIM-III) are designated as supplemental, highly recommended and, of those, three (10MWT, 2MWT, 6MWT) evaluated gait. Given that many rehabilitation technologies aim to improve functional ability beyond gait, there is a clear need for robust COAs to evaluate endpoints other than gait in SCI. The non-gait COA with NINDS CDE designation of supplemental, highly recommend is the SCIM-III, which has known limitations, especially with respect to ceiling and floor effects [4, 21–23] and inability to distinguish between recovery and compensation [23, 24]. Until recently [25], the SCIM-III lacked standardized guidelines for set-up and administration, which led to variability in administration and scoring [18, 19]. This variability poses a risk to the reliability of a clinical trial and may result in an overestimation or underestimation of function [25]. Implementing the standardization guidelines [26] in studies evaluating therapeutic effects is recommended.

To determine which COAs were able to differentiate between recovery of function and compensation, the team reviewed literature to identify COAs that may distinguish recovery vs. compensation [4, 27–29], considered the construct, items, and response scale for each COA, and obtained team consensus. CUE-T, GRASSP, and SCI-FAI were determined as able to distinguish between recovery and compensation due to their consideration of and ability to identify compensations during their administration process and/or response scale [4, 5]. The number of COAs that can distinguish between recovery of function and compensatory movement in SCI are limited. Those that can differentiate are narrow in measurement constructs. The measurement constructs of the GRASSP and CUE-T are sensorimotor function of the hand (wrist and fingers/thumb) and upper limb function (shoulder to hand), respectively, while the SCI-FAI’s concept of interest is ambulation and balance. Further, the GRASSP and CUE-T use an ordinal scale with the SCI-FAI having items that are nominal, ordinal, and ratio. Given that these measurement properties are not ideal for precise measurements, these COAs have inherent limitations for measuring therapeutic effects.

Integrative reviews, although less rigorous than systematic reviews, are more comprehensive given the inclusion of studies that are not randomized controlled trials. This allowed for the inclusion of a wide variety of COAs with constructs measuring motor and physical functioning. However, the inclusion of studies with different methodologies and study designs may have led to issues with inaccuracy, bias, or rigor [30, 31].

When extracting data, we only reported on the entire COA rather than on each separate subtest/subscale. If a study only used certain domains or subtests of a COA (e.g., self-care of SCIM I-III), this nuance was not captured in our extraction. Moreover, the mode of administration reported by the studies was what was presented in the study results. For example, if a study reported the SCIM I-III mode of administration as PRO, then that is what was documented when extracting and organizing study data, even though the SCIM I-III may be administered as either PerfO or PRO. If the COA was known to have multiple modes of administration and the author did not report which mode was used, then it was marked as “not reported”.

To obtain information about the measurement construct or response categories for COAs, the Rehab Measures Database (RMD) and Physiopedia were used as primary resources. If these databases did not have the information, we used peer-reviewed articles or Stroke Engine. When reporting on COAs, it is imperative that studies include information about the COA, such as measurement construct and response categories, and specify how the COA was administered and scored (e.g., mode of administration, total score versus subscale scores). This is important when considering study outcomes and for study replication.

### Limitations

There are several limitations of this study that may have impacted the comprehensiveness and accuracy of our search. Our initial search strategy included specific companies and brand names to include keywords that are as specific as possible to the literature we were seeking to find. Because the list of specific company names for this search strategy was not comprehensive, some manuscripts may have been missing. Further, we used the NINDS CDE designation as the primary guide to examine the COA and the REHAB Database, Physiopedia, Stroke engine and published studies to understand the measurement construct and response categories of the COAs. There are additional resources that we did not use that may have provided additional/different information about the COA. Lastly, if the team was uncertain about how to proceed with certain articles, consensus was obtained. These consensus positions may not be entirely accurate.

### Implications and recommendations

FES and exoskeletons are rehabilitation technologies that provide direct functional benefits when they are used, with potential therapeutic effects when they are not used. These therapeutic effects may be due to motor recovery, compensation, or a combination of both [4, 5, 32]. Thus, when therapeutic effect is of interest in a study of FES or exoskeletons it is imperative to select COAs that can differentiate motor recovery from compensatory strategies. Of the 38 COAs used in the studies included in this review, only three (CUE-T, GRASSP, and SCI-FAI) either disallowed or accounted for compensation of movement; all are designated as supplemental within the NINDS SCI CDE structure.

The results of this review catalyzed several suggestions for the field. First, it is suggested that all SCI studies, not only ones funded by NIH, consider the NINDS SCI CDE designations when selecting COAs. Second, when reporting on COAs that are validated as both PerfO and PRO, studies should make explicit the mode of administration (e.g., PRO, PerfO) used. Finally, most of the SCI COA do not account for compensatory movement strategies, which can lead to both underestimating and overestimating the therapeutic effects of interventions that target motor recovery. When recovery of movement is a study endpoint, prudence is warranted when utilizing COAs that do not account for compensation.

## Conclusion

There are four COAs that are commonly used to evaluate the therapeutic effects of FES and exoskeletons on gait; three are ratio (10MWT, 2MWT, 6MWT) and one is ordinal (WISCI-II) levels of measurement; each are designated as supplemental, highly recommended (10MWT, 2MWT, 6MWT) or supplemental (WISCI-II) by NINDS SCI CDE structure. Robust COA to measure the therapeutic effects of FES and exoskeletons on upper extremity and overall physical functioning are lacking. Those that do exist are either narrow in scope, have known limitations, and/or do not differentiate between recovery and compensation. There is an opportunity to expand our measurement toolkit with more robust and precise COAs that disallow and/or account for compensatory movement.

## Supplementary information

Appendix 1

## Data Availability

All data generated or analyzed during this study are included in this published article [and its supplementary information files].
