# Supplementary material for: Evaluating therapeutic effects of exoskeletons and FES in SCI: integrative review of the literature
Source: Spinal Cord. 2025 May 29;63(7):323–32. doi: 10.1038/s41393-025-01085-x (PMC12237692; doi:10.1038/s41393-025-01085-x)
Supplement: Supplementary file 1 — Appendix 1 [file 41393_2025_1085_MOESM1_ESM.docx]

1. Aach M, Cruciger O, Sczesny-Kaiser M, et al. Voluntary driven exoskeleton as a new tool for rehabilitation in chronic spinal cord injury: a pilot study. *Spine J: Official J N Am Spine Society*. 2014;14(12):2847-2853. doi:[10.1016/j.spinee.2014.03.042](https://doi.org/10.1016/j.spinee.2014.03.042)

2. Bach Baunsgaard C, Vig Nissen U, Katrin Brust A, et al. Gait training after spinal cord injury: safety, feasibility and gait function following 8 weeks of training with the exoskeletons from Ekso Bionics. *Spinal Cord*. 2018;56(2):106-116. doi:10.1038/s41393-017-0013-7

3. Bailey SN, Hardin EC, Kobetic R, Boggs LM, Pinault G, Triolo RJ. Neurotherapeutic and neuroprosthetic effects of implanted functional electrical stimulation for ambulation after incomplete spinal cord injury. *J Rehabil Res Dev*. 2010;47(1):7-16. doi:10.1682/jrrd.2009.03.0034

4. Baunsgaard CB, Nissen UV, Brust AK, et al. Exoskeleton gait training after spinal cord injury: an exploratory study on secondary health conditions. *J Rehabil Med*. 2018;50(9):806-813. doi:10.2340/16501977-2372

5. Brinkemper A, Aach M, Grasmücke D, et al. Improved physiological gait in acute and chronic SCI patients after training with wearable cyborg hybrid assistive limb. *Front Neurorobotics*. 2021;15:723206. doi:10.3389/fnbot.2021.723206

6. Chang S-H, Afzal T; TIRR SCI Clinical Exoskeleton Group, Berliner J, Francisco GE. Exoskeleton-assisted gait training to improve gait in individuals with spinal cord injury: a pilot randomized study. *Pilot Feasibility Stud*. 2018;4:62. doi:10.1186/s40814-018-0247-y

7. Cruciger O, Schildhauer TA, Meindl RC, et al. Impact of locomotion training with a neurologic controlled hybrid assistive limb (HAL) exoskeleton on neuropathic pain and health related quality of life (HRQoL) in chronic SCI: a case study. *Disabil Rehabil Assist Technol*. 2016;11(6):529-534. doi:10.3109/17483107.2014.981875

8. Duffell LD, Paddison S, Alahmary AF, Donaldson N, Burridge J. The effects of FES cycling combined with virtual reality racing biofeedback on voluntary function after incomplete SCI: a pilot study. *J Neuroeng Rehabil*. 2019;16(1):149. doi:10.1186/s12984-019-0619-4

9. Esclarín-Ruz A, Alcobendas-Maestro M, Casado-Lopez R, et al. A comparison of robotic walking therapy and conventional walking therapy in individuals with upper versus lower motor neuron lesions: a randomized controlled trial. *Arch Phys Med Rehabil*. 2014;95(6):1023-1031. doi:10.1016/j.apmr.2013.12.017

10. Field-Fote EC. Combined use of body weight support, functional electric stimulation, and treadmill training to improve walking ability in individuals with chronic incomplete spinal cord injury. *Arch Phys Med Rehabil*. 2001;82(6):818-824. doi:10.1053/apmr.2001.23752

11. Fleerkotte BM, Koopman B, Buurke JH, van Asseldonk EHF, van der Kooij H, Rietman JS. The effect of impedance-controlled robotic gait training on walking ability and quality in individuals with chronic incomplete spinal cord injury: an explorative study. *J Neuroeng Rehabil*. 2014;11:26. doi:10.1186/1743-0003-11-26

12. Francisco GE, Yozbatiran N, Berliner J, et al. Robot-assisted training of arm and hand movement shows functional improvements for incomplete cervical spinal cord injury. *Am J Phys Med Rehabil*. 2017;96(10 Suppl 1):S171-S177. doi:10.1097/PHM.0000000000000815

13. Granat MH, Ferguson AC, Andrews BJ, Delargy M. The role of functional electrical stimulation in the rehabilitation of patients with incomplete spinal cord injury – observed benefits during gait studies. *Paraplegia*. 1993;31(4):207-215. doi:10.1038/sc.1993.39

14. Grasmücke D, Zieriacks A, Jansen O, et al. Against the odds: what to expect in rehabilitation of chronic spinal cord injury with a neurologically controlled Hybrid Assistive Limb exoskeleton: a subgroup analysis of 55 patients according to age and lesion level. *Neurosurg Focus*. 2017;42(5):E15. doi:10.3171/2017.2.FOCUS171

15. Harvey LA, Dunlop SA, Churilov L, Galea MP, Spinal Cord Injury Physical Activity (SCIPA) Hands On Trial Collaborators. Early intensive hand rehabilitation is not more effective than usual care plus one-to-one hand therapy in people with sub-acute spinal cord injury ('Hands On’): a randomised trial. *J Physiother*. 2016;62(2):88-95. doi:10.1016/j.jphys.2016.02.013

16. Harvey LA, Fornusek C, Bowden JL, et al. Electrical stimulation plus progressive resistance training for leg strength in spinal cord injury: a randomized controlled trial. *Spinal Cord*. 2010;48(7):570-575. doi:10.1038/sc.2009.191

17. Hesse S, Werner C, Bardeleben A. Electromechanical gait training with functional electrical stimulation: case studies in spinal cord injury. *Spinal Cord*. 2004;42(6):346-352. doi:10.1038/sj.sc.3101595

18. Hitzig SL, Craven BC, Panjwani A, et al. Randomized trial of functional electrical stimulation therapy for walking in incomplete spinal cord injury: effects on quality of life and community participation. *Top Spinal Cord Inj Rehabil*. 2013;19(4):245-258. doi:10.1310/sci1904-245

19. Hoffman L, Field-Fote E. Effects of practice combined with somatosensory or motor stimulation on hand function in persons with spinal cord injury. *Top Spinal Cord Inj Rehabil*. 2013;19(4):288-299. doi:10.1310/sci1904-288

20. Houston DJ, Lee JW, Unger J, Masani K, Musselman KE. Functional electrical stimulation plus visual feedback balance training for standing balance performance among individuals with incomplete spinal cord injury: a case series. *Front Neurol*. 2020;11:680. doi:10.3389/fneur.2020.00680

21. Jang Y-C, Park H-K, Han J-Y, Choi IS, Song M-K. Cardiopulmonary function after robotic exoskeleton-assisted over-ground walking training of a patient with an incomplete spinal cord injury: case report. *Med (Baltim)*. 2019;98(50):e18286. doi:10.1097/MD.0000000000018286

22. Jansen O, Grasmuecke D, Meindl RC, et al. Hybrid assistive limb exoskeleton HAL in the rehabilitation of chronic spinal cord injury: proof of concept; the results in 21 patients. *World Neurosurg*. 2018;110:e73-e78. doi:10.1016/j.wneu.2017.10.080

23. Jansen O, Schildhauer TA, Meindl RC, et al. Functional outcome of neurologic-controlled HAL-exoskeletal neurorehabilitation in chronic spinal cord injury: a pilot with one year treatment and variable treatment frequency. *Global Spine J*. 2017;7(8):735-743. doi:10.1177/2192568217713754

24. Jones ML, Evans N, Tefertiller C, et al. Activity-based therapy for recovery of walking in individuals with chronic spinal cord injury: results from a randomized clinical trial. *Arch Phys Med Rehabil*. 2014;95(12):2239-46.e2. doi:10.1016/j.apmr.2014.07.400

25. Kapadia N, Masani K, Catharine Craven B, et al. A randomized trial of functional electrical stimulation for walking in incomplete spinal cord injury: effects on walking competency. *J Spinal Cord Med*. 2014;37(5):511-524. doi:10.1179/2045772314Y.0000000263

26. Kapadia N, Zivanovic V, Popovic MR. Restoring voluntary grasping function in individuals with incomplete chronic spinal cord injury: pilot study. *Top Spinal Cord Inj Rehabil*. 2013;19(4):279-287. doi:10.1310/sci1904-279

27. Khan AS, Livingstone DC, Hurd CL, et al. Retraining walking over ground in a powered exoskeleton after spinal cord injury: a prospective cohort study to examine functional gains and neuroplasticity. *J Neuroeng Rehabil*. 2019;16(1):145. doi:10.1186/s12984-019-0585-x

28. Kumru H, Benito-Penalva J, Valls-Sole J, et al. Placebo-controlled study of rTMS combined with Lokomat® gait training for treatment in subjects with motor incomplete spinal cord injury. *Exp Brain Res*. 2016;234(12):3447-3455. doi:10.1007/s00221-016-4739-9

29. Kumru H, Murillo N, Benito-Penalva J, Tormos JM, Vidal J. Transcranial direct current stimulation is not effective in the motor strength and gait recovery following motor incomplete spinal cord injury during Lokomat® gait training. *Neurosci Lett*. 2016;620:143-147. doi:10.1016/j.neulet.2016.03.056

30. Lu Z, Tong K-Y, Shin H, Stampas A, Zhou P. Robotic hand-assisted training for spinal cord injury driven by myoelectric pattern recognition: a case report. *Am J Phys Med Rehabil*. 2017;96(10 Suppl 1):S146-S149. doi:10.1097/PHM.0000000000000798

31. Momeni K, Ramanujam A, Garbarini EL, Forrest GF. Multi-muscle electrical stimulation and stand training: effects on standing. *J Spinal Cord Med*. 2019;42(3):378-386. doi:10.1080/10790268.2018.1432311

32. Okawara H, Sawada T, Matsubayashi K, et al. Gait ability required to achieve therapeutic effect in gait and balance function with the voluntary driven exoskeleton in patients with chronic spinal cord injury: a clinical study. *Spinal Cord*. 2020;58(5):520-527. doi:10.1038/s41393-019-0403-0

33. Postans NJ, Hasler JP, Granat MH, Maxwell DJ. Functional electric stimulation to augment partial weight-bearing supported treadmill training for patients with acute incomplete spinal cord injury: a pilot study. *Arch Phys Med Rehabil*. 2004;85(4):604-610. doi:10.1016/j.apmr.2003.08.083

34. Powell ES, Carrico C, Raithatha R, Salyers E, Ward A, Sawaki L. Transvertebral direct current stimulation paired with locomotor training in chronic spinal cord injury: a case study. *NeuroRehabilitation*. 2016;38(1):27-35. doi:10.3233/NRE-151292

35. Rahimi M, Torkaman G, Ghabaee M, Ghasem-Zadeh A. Advanced weight-bearing mat exercises combined with functional electrical stimulation to improve the ability of wheelchair-dependent people with spinal cord injury to transfer and attain independence in activities of daily living: a randomized controlled trial. *Spinal Cord*. 2020;58(1):78-85. doi:10.1038/s41393-019-0328-7

36. Raithatha R, Carrico C, Powell ES, et al. Non-invasive brain stimulation and robot-assisted gait training after incomplete spinal cord injury: a randomized pilot study. *NeuroRehabilitation*. 2016;38(1):15-25. doi:10.3233/NRE-151291

37. Rodionov A, Savolainen S, Kirveskari E, Mäkelä JP, Shulga A. Restoration of hand function with long-term paired associative stimulation after chronic incomplete tetraplegia: a case study. *Spinal Cord Ser Cases*. 2019;5:81. doi:10.1038/s41394-019-0225-5

38. Sawada T, Okawara H, Matsubayashi K, et al. Influence of body weight-supported treadmill training with voluntary-driven exoskeleton on the quality of life of persons with chronic spinal cord injury: a pilot study. *Int J Rehabil Res*. 2021;44(4):343-349. doi:10.1097/MRR.0000000000000496

39. Sharif H, Gammage K, Chun S, Ditor D. Effects of FES-ambulation training on locomotor function and health-related quality of life in individuals with spinal cord injury. *Top Spinal Cord Inj* *Rehabil*. 2014;20(1):58-69. doi:10.1310/sci2001-58

40. Soma Y, Kubota S, Kadone H, et al. Hybrid assistive limb functional treatment for a patient with chronic incomplete cervical spinal cord injury. *Int Med Case Rep J*. 2021;Volume 14:413-420. doi:10.2147/IMCRJ.S306558

41. Sørensen L, Månum G. A single-subject study of robotic upper limb training in the subacute phase for four persons with cervical spinal cord injury. *Spinal Cord Ser Cases*. 2019;5:29. doi:10.1038/s41394-019-0170-3

42. Stampacchia G, Olivieri M, Rustici A, D’Avino C, Gerini A, Mazzoleni S. Gait rehabilitation in persons with spinal cord injury using innovative technologies: an observational study. *Spinal Cord*. 2020;58(9):988-997. doi:10.1038/s41393-020-0454-2

43. Street T, Singleton C. A clinically meaningful training effect in walking speed using functional electrical stimulation for motor-incomplete spinal cord injury. *J Spinal Cord Med*. 2018;41(3):361-366. doi:10.1080/10790268.2017.1392106

44. Tamburella F, Tagliamonte NL, Pisotta I, et al. Neuromuscular controller embedded in a powered ankle exoskeleton: effects on gait, clinical features and subjective perspective of incomplete spinal cord injured subjects. *IEEE Trans Neural Syst Rehabil Eng*. 2020;28(5):1157-1167. doi:10.1109/TNSRE.2020.2984790

45. Thorsen R, Dalla Costa D, Chiaramonte S, et al. A noninvasive neuroprosthesis augments hand grasp force in individuals with cervical spinal cord injury: the functional and therapeutic effects. *Sci World J*. 2013;2013:836959. doi:10.1155/2013/836959

46. Thrasher TA, Flett HM, Popovic MR. Gait training regimen for incomplete spinal cord injury using functional electrical stimulation. *Spinal Cord*. 2006;44(6):357-361. doi:10.1038/sj.sc.3101864

47. Tsai C-Y, Asselin PK, Hong E, et al. Exoskeletal-assisted walking may improve seated balance in persons with chronic spinal cord injury: a pilot study. *Spinal Cord Ser Cases*. 2021;7(1):20. doi:10.1038/s41394-021-00384-8

48. Tsai C-Y, Delgado AD, Weinrauch WJ, et al. Exoskeletal-assisted walking during acute inpatient rehabilitation leads to motor and functional improvement in persons with spinal cord injury: a pilot study. *Arch Phys Med Rehabil*. 2020;101(4):607-612. doi:10.1016/j.apmr.2019.11.010

49. Venugopalan L, Taylor PN, Cobb JE, Swain ID. TetraGrip - a four channel upper limb FES device for people with C5/C6 tetraplegia: device design and clinical outcome. *J Med Eng Technol*. 2020;44(1):38-44. doi:10.1080/03091902.2020.1713239

50. Wieler M, Stein RB, Ladouceur M, et al. Multicenter evaluation of electrical stimulation systems for walking. *Arch Phys Med Rehabil*. 1999;80(5):495-500. doi:10.1016/s0003-9993(99)90188-0

51. Yildirim MA, Öneş K, Gökşenoğlu G. Early term effects of robotic assisted gait training on ambulation and functional capacity in patients with spinal cord injury. *Turk J Med Sci*. 2019;49(3):838-843. doi:10.3906/sag-1809-7

52. Yozbatiran N, Berliner J, Boake C, O’Malley MK, Kadivar Z, Francisco GE. Robotic training and clinical assessment of forearm and wrist movements after incomplete spinal cord injury: a case study. *IEEE Int Conf Rehabil Robot*. 2011;2011:5975425. doi:10.1109/ICORR.2011.5975425

53. Yozbatiran N, Berliner J, O’Malley MK, et al. Robotic training and clinical assessment of upper extremity movements after spinal cord injury: a single case report. *J Rehabil Med*. 2012;44(2):186-188. doi:10.2340/16501977-0924

54. Yozbatiran N, Keser Z, Davis M, et al. Transcranial direct current stimulation (tDCS) of the primary motor cortex and robot-assisted arm training in chronic incomplete cervical spinal cord injury: a proof of concept sham-randomized clinical study. *NeuroRehabilitation*. 2016;39(3):401-411. doi:10.3233/NRE-161371

55. Zariffa J, Kapadia N, Kramer JLK, et al. Effect of a robotic rehabilitation device on upper limb function in a sub-acute cervical spinal cord injury population. *IEEE Int Conf Rehabil Robot*. 2011;2011:5975400. doi:10.1109/ICORR.2011.5975400

56. Zhou R, Alvarado L, Ogilvie R, Chong SL, Shaw O, Mushahwar VK. Non-gait-specific intervention for the rehabilitation of walking after SCI: role of the arms. *J Neurophysiol*. 2018;119(6):2194-2211. doi:10.1152/jn.00569.2017
